# A diamidobenzimidazole STING agonist protects against SARS-CoV-2 infection

**DOI:** 10.1126/sciimmunol.abi9002

**Published:** 2021-05-18

**Authors:** Fiachra Humphries, Liraz Shmuel-Galia, Zhaozhao Jiang, Ruth Wilson, Philip Landis, Sze-Ling Ng, Krishna-Mohan Parsi, Rene Maehr, John Cruz, Angel Morales-Ramos, Joshi M. Ramanjulu, John Bertin, G. Scott Pesiridis, Katherine A. Fitzgerald

**Affiliations:** 1 Program in Innate Immunity, Department of Medicine, University of Massachusetts Medical School, Worcester, MA 01605, USA.; 2 Innate Immunity Research Unit. GlaxoSmithKline, Collegeville, PA, USA.; 3 Program in molecular medicine, University of Massachusetts Medical School, Worcester, MA 01605, USA.; 4 Department of pathology, University of Massachusetts Medical School, Worcester, MA 01605, USA.

## Abstract

Coronaviruses are a family of RNA viruses that cause acute and chronic diseases of the upper and lower respiratory tract in humans and other animals. SARS-CoV-2 is a recently emerged coronavirus that has led to a global pandemic causing a severe respiratory disease known as COVID-19 with significant morbidity and mortality worldwide. The development of antiviral therapeutics are urgently needed while vaccine programs roll out worldwide. Here we describe a diamidobenzimidazole compound, diABZI-4, that activates STING and is highly effective in limiting SARS-CoV-2 replication in cells and animals. diABZI-4 inhibited SARS-CoV-2 replication in lung epithelial cells. Administration of diABZI-4 intranasally before or even after virus infection conferred complete protection from severe respiratory disease in K18-ACE2-transgenic mice infected with SARS-CoV-2. Intranasal delivery of diABZI-4 induced a rapid short-lived activation of STING, leading to transient proinflammatory cytokine production and lymphocyte activation in the lung associated with inhibition of viral replication. Our study supports the use of diABZI-4 as a host-directed therapy which mobilizes antiviral defenses for the treatment and prevention of COVID-19.

## Teaser

Pharmacological activation of STING protects against SARS-CoV-2 infection.

## INTRODUCTION

As of January 2021, the recently emerged severe-acute respiratory syndrome coronavirus 2 has led to over 2 million deaths and over 100 million infections globally (*1*). SARS-CoV-2 is a member of the *Coronaviridae* family of viruses. Respiratory infections with SARS-CoV-2 can result in asymptomatic, mild or severe forms of a disease known as COVID-19. More severe cases of COVID-19 result in death due to acute respiratory distress syndrome and damage to the alveolar lumen (*2*). Currently, there are few treatment options for COVID-19 patients. The antiviral RNA-dependent polymerase inhibitor remdesivir reduces length of hospitalization and deaths from COVID-19 (*3*). In addition, the steroid dexamethasone has also been approved for use in severe COVID-19 (*4*). To date numerous efficacious vaccines have been developed and rolled out (*5*, *6*). Despite these advances additional antiviral therapeutics will be required for the treatment of future endemic infections. An ongoing global effort is now underway to identify and develop new antiviral and anti-inflammatory therapeutics to reduce COVID-19 related hospitalizations and deaths.

The ER-resident adaptor protein Stimulator of Interferon Genes is a key signaling molecule that is activated following cytosolic DNA detection. Cyclic GMP-AMP synthase is an innate immune sensor of cytosolic DNA. Upon DNA binding cGAS converts ATP and GTP into the cyclic dinucleotide cGAMP, which in turn binds and activates STING (*7*). Conformational changes in STING lead to C-terminal phosphorylation of STING, dimerization, oligomerization and subsequent activation of autophagy, NF-κB, IRF3 and transcription of proinflammatory cytokines and type I IFNs (*8*–*10*). Activation of STING can elicit a potent anti-tumor response and the use of STING agonists in oncology alone or in combination with checkpoint blockade is an emerging therapeutic area (*11*–*13*). A recent study has identified a new class of STING agonists with systemic in vivo activity. Diamidobenzimidazole based compounds are potent, specific activators of STING and possess superior stability, tissue penetrance and potency over traditional cyclic dinucleotide STING agonists (*14*). While the therapeutic applications of STING agonists have been reported for use in oncology, the antiviral potential of STING agonists remains underexplored. Given the potent type I IFN response induced by diABZI compounds, we hypothesized that pharmacological activation of STING may elicit protection from SARS-CoV-2 infection.

## RESULTS

### diABZI-4 activates STING

**Fig. 1:**
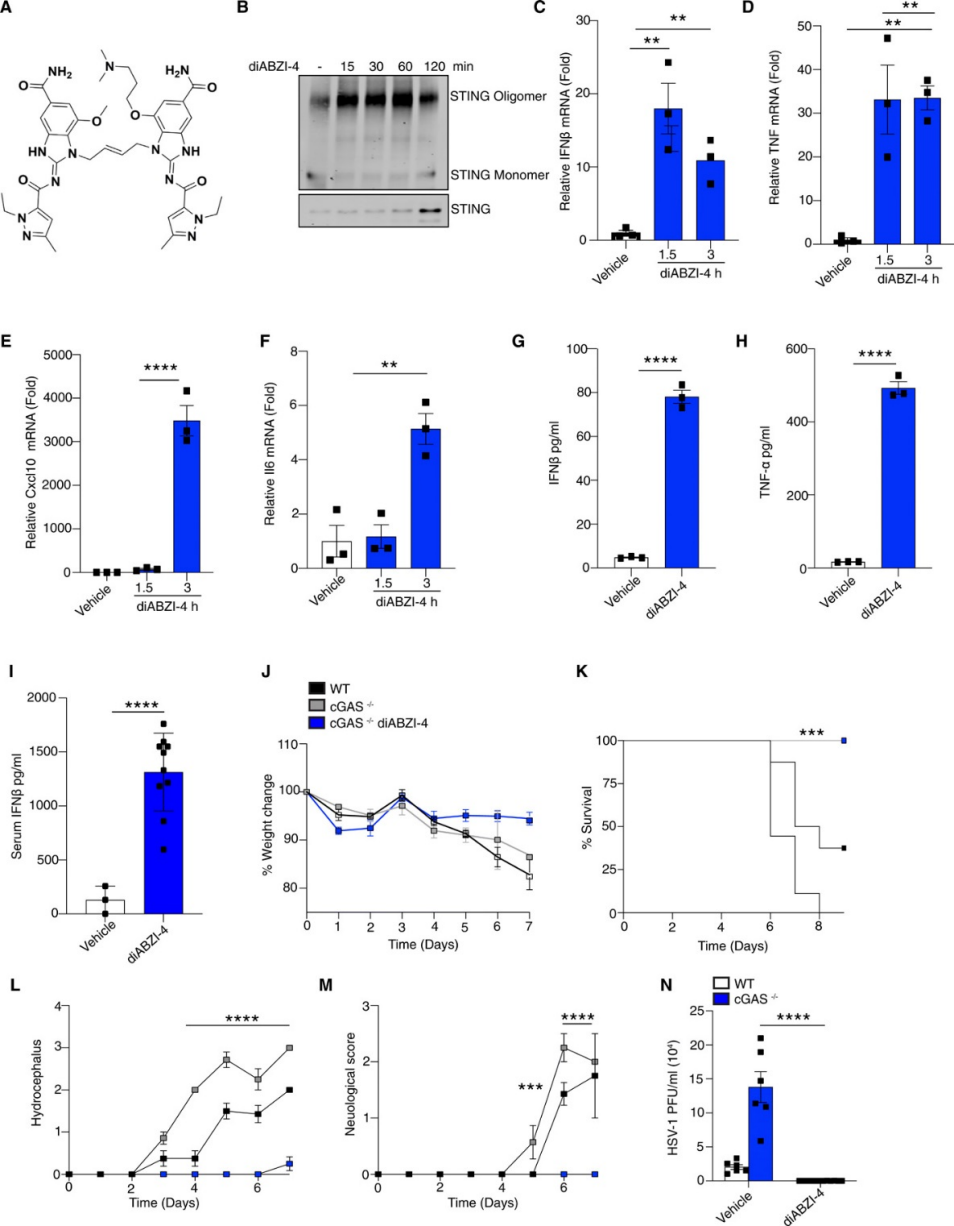
**diABZI-4 promotes potent STING activation and protection from HSE (A)** Structure of diABZI-4. **(B)** Native immunoblot of STING dimerization in CD14^+^ human monocytes treated with 0.1 μM of diABZI-4 for the indicated times. (C-F) QPCR analysis of IFNβ (C), TNF-α (D), CXCL10 (E) and IL-6 (F) mRNA expression in CD14^+^ human monocytes treated with 0.1 μM of diABZI-4 for the indicated times. IFNβ (G) and TNF-α (H) ELISA in CD14^+^ human monocytes treated with 0.1 μM of diABZI-4 for the indicated times. **(I)** Serum IFNβ levels from WT mice 3 hours after intraperitoneal injection with 1mg/kg diABZI-4 (*n=10*) or PBS (*n=3*). **(J-M)** Weight loss (J) survival (K) hydrocephalus (L) and neurological symptom scores of WT (*n=8*) and cGAS^−/−^ (*n=7-8*) mice infected in the cornea with 2x10^5^ PFU of HSV-1 McKrae with a 1 hour retro-orbital pre-treatment of vehicle control or 0.5mg/kg of diABZI-4. C-H are pooled data from three independent experiments, error bars show means ± SEM. B, representative experiment. I, data points indicate individual mice. J-M, data points indicate mean ± SD of each group. **, *P*<0.01; ***, *P*<0.001; ****, *P*<0.0001 (B-F one way ANOVA. G-I Student’s *t* test. J, L-M 2-way ANOVA. K, Mantel-Cox).

To evaluate the antiviral properties of diABZI compounds we utilized a new diABZI compound, diABZI-4 (**Fig. 1A****, supplementary methods**). diABZI-4 has a more favorable solubility profile for in vivo studies in comparison to the previously reported diABZI-3 (*14*). diABZI-4 induced oligomerization of STING (**Fig. 1B**), transcription of *IFNB1*, *CXCL10*, *TNF* and *IL6* (**Fig. 1C-F**) and the secretion of IFN-β (**Fig. 1G**) and TNF-α (**Fig. 1H**) in primary human CD14+ monocytes. Similarly, diABZI-4 potently activated STING in murine cells. Treatment of bone marrow derived macrophages (BMDMs) with diABZI-4 resulted in oligomerization of STING (**Fig. S1A**), phosphorylation of IRF3, TBK1, p65 and autophagy (LC3 conversion) (**Fig. S1B**). Treatment of BMDMs with diABZI-4 also induced the expression (**Fig. S1C-F**) and secretion (**Fig. S1G-J**) of IFN-β, CXCL10, TNF-α and IL-6. In vivo administration of diABZI-4 via intraperitoneal injection also induced the production of IFN-β (**Fig. 1I**).

### diABZI-4 protects against herpes simplex encephalitis

To evaluate the potential antiviral effects of systemically administered diABZI-4, we first tested the efficacy of diABZI-4 in a mouse model of herpes simplex encephalitis. cGAS- and STING-deficient mice are highly susceptible to acute HSV-1 infection (*15*). Indeed, both cGAS and STING-deficient mice displayed increased weight loss, hydrocephalus and decreased survival following corneal infection with a neuroinvasive strain of HSV-1 (**Fig. S2A-F**). Thus, we hypothesized that diABZI-4 may compensate for cGAS deficiency and confer protection from HSE through activation of STING. Treatment of cGAS^−/−^ mice with a single dose of diABZI-4 delivered via retro-orbital injection resulted in complete protection from HSE (**Fig. 1J-M**). cGAS^−/−^ mice receiving diABZI-4 were protected from HSV-1–induced weight loss (**Fig. 1J**), lethality (**Fig. 1K**), hydrocephalus (**Fig. 1L**) and symptoms of neurological disease (**Fig. 1M**). In addition, cGAS^−/−^ mice receiving diABZI-4 had a significant reduction in HSV-1 titers in brain tissue **(****Fig. 1N****).**

### diABZI-4 inhibits SARS-CoV-2 replication

**Fig. 2:**
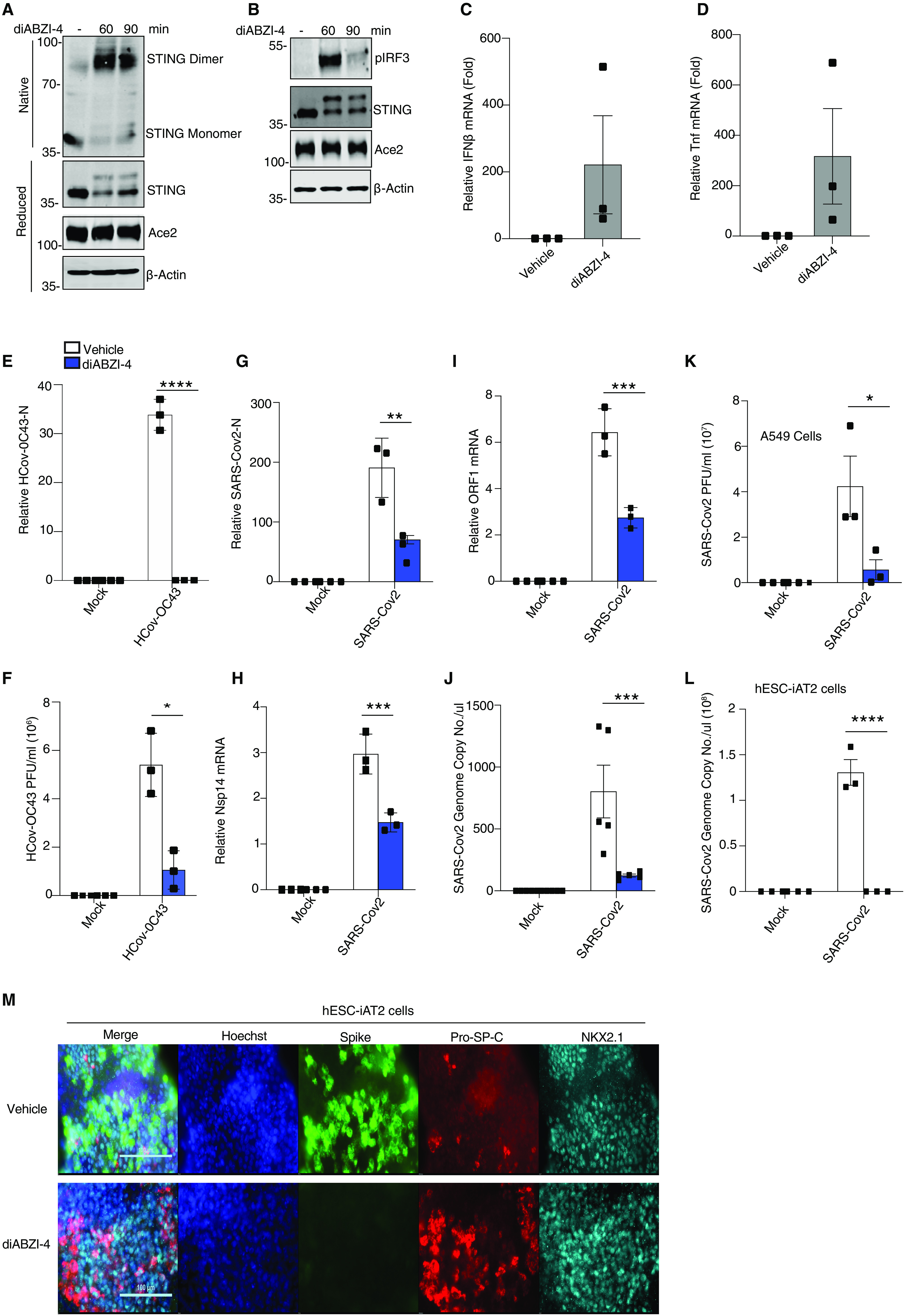
**diABZI-4 inhibits SARS-CoV-2 replication in lung epithelial cells. (A)** Native immunoblot of STING dimerization from ACE-A549 cells treated with 0.1 μM of diABZI-4 for the indicated times. **(B)** Immunoblot analysis of p-IRF3, STING, ACE2 and β-actin from ACE2-A549 cells treated with 0.1 μM of diABZI-4 for the indicated times. **(C-D)** QPCR analysis of IFNβ (C) and TNF-α (D) mRNA expression in ACE2-A549 cells treated with 0.1 μM of diABZI-4 for 1.5 hours. **(E)** QPCR analysis of HCoV-OC43-N mRNA from ACE2-A549 cells infected with HCoV-OC43 0.1 MOI for 24 hours with or without pre-treatment of 0.1 μM diABZI-4. **(F)** TCID50 assay of conditioned medium from ACE2-A549 cells treated as in E for 48 hours. **(G-I)** QPCR analysis of SARS-CoV-2-N (G), Nsp14 (H) and ORF1 (I) mRNA from ACE2-A549 cells infected with SARS-CoV-2 0.1 MOI for 24 hours with or without pre-treatment of 0.1 μM diABZI-4. **(J)** Genome copy number analysis on conditioned medium from ACE2-A549 cells treated as in I. **(K)** TCID50 assay of conditioned medium from ACE2-A549 cells infected with SARS-CoV-2 0.1 MOI for 48 hours with or without pre-treatment of 0.1 μM diABZI-4. (**L**) Genome copy number analysis on ACE2-hESC-iAT2 cells infected with SARS-CoV-2 0.1 MOI for 72 hours with or without pre-treatment of 0.1 μM diABZI-4. **(M)** Immunofluorescence analysis of SARS-CoV-2 spike, Pro-SP-C and NKX2.1 in hESC-iAT2 cells infected with SARS-CoV-2 0.1 MOI for 72 hours with or without pre-treatment of 0.1 μM diABZI-4 A-B, M representative experiment. C-L pooled data from 3-5 independent experiments. *, *P*<0.05; ***, *P*<0.001; ****, *P*<0.0001 (E-K, two-way ANOVA). Error bars show means ± SEM.

Based on these findings we next assessed if diABZI-4 could also confer protection against RNA viruses. We focused on human coronavirus OC-43 (HCoV-OC43) and SARS-CoV-2. SARS-CoV-2 infects lung epithelial cells via the ACE2 receptor (*16*). Therefore, we first monitored STING activation in ACE2-expressing A549 cells treated with diABZI-4 which resulted in the time-dependent dimerization of STING (**Fig. 2A**), phosphorylation of IRF3 (**Fig. 2B**) and expression of *IFNB1* (**Fig. 2C**) and *TNF* (**Fig. 2D**). ACE2-A549 cells were highly permissive to HCoV-OC43 and SARS-CoV-2 infection (*2*). Pre-treatment of ACE2-A549 cells with diABZI-4 prior to infection with HCoV-OC43 inhibited HCoV-OC43 replication when compared to vehicle-treated cells (**Fig. 2E-F**). Similarly, pre-treatment with diABZI-4 inhibited SARS-CoV-2 gene expression and replication in infected cells when compared to vehicle-treated cells (**Fig. 2G-K**). Given the strong effects diABZI-4 had on SARS-CoV-2 replication in hACE2-A549 cells we next evaluated its effects on SARS-CoV-2 in a more physiologically relevant lung alveolar cell system. Embryonic stem cell-derived induced alveolar type II (iAT2) cells were cultured in 3D at air-liquid interface (ALI). Pre-treatment of iAT2 cells with diABZI-4 inhibited SARS-CoV-2 replication. (**Fig. 2L-M**). Two other studies have also shown diABZI molecules impair SARS-CoV-2 replication in lung epithelial cells (*17*, *18*)

### diABZI-4 protects against SARS-CoV-2 infection

**Fig. 3:**
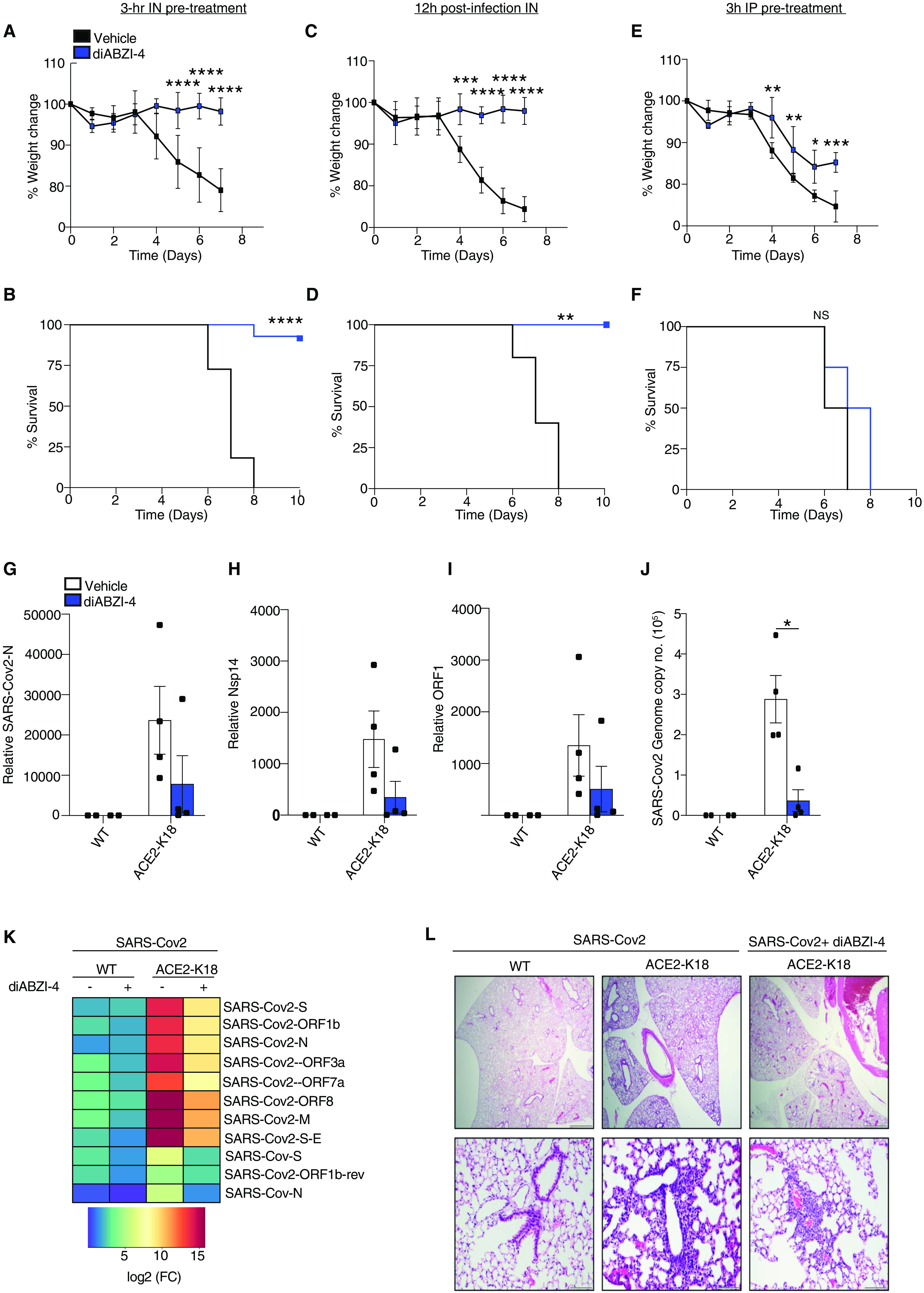
**diABZI-4 protects against SARS-CoV-2 infection in hACE2 transgenic mice** Survival (A) and weight loss (B) of K18-ACE2 transgenic mice infected intranasally with SARS-CoV-2 (2.5x10^4^ PFU/mouse) with a 3 hours intranasal pre-treatment of 0.5mg/kg diABZI-4 (*n=13*) or vehicle control (*n=11*). **(C-D)** Survival (C) and weight loss (D) of K18-ACE2^+/−^ transgenic mice infected intranasally with SARS-CoV-2 (2.5x10^4^ PFU/mouse) followed by intranasal treatment with 0.5mg/kg of diABZI-4 (*n=5*) or vehicle control (*n=5*) 12 hours post-infection. **(E-F)** Survival (E) and weight loss (F) of K18-ACE2 transgenic mice infected intranasally with SARS-CoV-2 (2.5x10^4^ PFU/mouse) with a 3 hours intraperitoneal injection of 0.5mg/kg diABZI-4 (*n=4*) or vehicle control (*n=4*). **(G-J)** QPCR analysis of SARS-CoV-2-N (G), Nsp14 (H) ORF1 (I) and genome copy number (J) in lung tissue of K18-ACE2 transgenic mice infected with SARS-CoV-2 (2.5x10^4^ PFU/mouse) for 48 hours with a 3 hours intranasal pre-treatment of 0.5mg/kg diABZI-4 (*n=4*) or vehicle control (*n=4*). **(K)** Heat-map of NanoString SARS-CoV-2 RNA transcripts in RNA extracted from SARS-CoV-2 infected K18-ACE2 mice treated intranasally with 0.5 mg/kg of diABZI-4 or vehicle control. Data presented as mean of calculated as fold reads in diABZI-4 treated over reads untreated mice (*n=2-3 per group*). **(L)** Representative images of H&E-stained lung sections from K18-ACE2 transgenic mice infected intranasally with SARS-CoV-2 (2.5x10^4^ PFU/mouse) with a 3 hours intraperitoneal injection of 0.5mg/kg diABZI-4 (*n=4*) or vehicle control (*n=4*) for 5 days. ***, *P*<0.0001, ****, *P*<0.00001. (A, C, E Mantel–Cox survival analysis). Error bars show means ± SEM.

Given that diABZI-4 strongly inhibited SARS-CoV-2 replication in lung epithelial cells we next determined if diABZI-4 could also prevent SARS-CoV-2 infection in vivo. SARS-CoV-2 cannot bind to murine ACE2; thus, a SARS-CoV-2 infection cannot be established in conventional laboratory mouse strains (*16*, *19*). K18-hACE2 transgenic mice (K18-ACE2), express human ACE2 under the control of the epithelial cell cytokeratin-18 (K18) promoter (*20*). Following intranasal inoculation with SARS-CoV-2, K18-ACE2 mice began to lose weight 4 days post-infection and die 7-8 days after infection (*21*, *22*). Thus, we utilized the K18-ACE2 mouse model to determine the effects of diABZI-4 on SARS-CoV-2 infection in vivo. K18-ACE2 mice inoculated intranasally with SARS-CoV-2 resulted in weight loss and lethality 8 days post-infection. However, K18-ACE2 mice administered a single intranasal dose of diABZI-4 were protected from SARS-CoV-2 induced weight loss (**Fig. 3A**) and lethality (**Fig, 3B**). Remarkably, diABZI-4 also protected mice from SARS-CoV-2 induced weight loss (**Fig. 3C**) and lethality (**Fig. 3D**) when administered 12 hours post-infection. Administration of the same dose of diABZI-4 via intraperitoneal injection failed to protect K18-ACE2 mice from SARS-CoV-2 infection (**Fig. 3E-F**) indicating that direct delivery of diABZI-4 to mucosal surfaces was required for its protective effects. diABZI-4 and diABZI-3 conferred comparable protection from SARS-CoV-2–induced weight loss (**Fig. S3A**) and lethality (**Fig. S3B**). A concentration of 0.25 mg/kg was required for complete protection from SARS-CoV-2–induced weight loss (**Fig. S3C**) and lethality (**Fig. S3D**). Given these striking effects we next assessed the effect of diABZI-4 on viral loads in the lung. Pre-treatment of K18-ACE2 mice with diABZI-4 resulted in a decrease in the expression of numerous SARS-CoV-2 genes including N-protein (**Fig. 3G**), Nsp14 (**Fig. 3H**) and ORF1 (**Fig. 3I**) in lung tissue collected 48 hours post-infection. Pre-treatment with diABZI-4 also resulted in a decrease in the genome copy number of SARS-CoV-2 when compared to lung tissue from K18-ACE2 mice treated with vehicle control **(****Fig. 3J****)**. NanoString analysis also demonstrated a significant decrease in SARS-CoV-2 RNA transcripts which correlated with a reduction in expression of a number of inflammatory genes in lung tissue from diABZI-4-treated K18-ACE2 mice indicative of enhanced, rapid viral clearance (**Fig. 3K****, Fig. S4A**). Analysis of hematoxylin and eosin (H&E)-stained lung sections from K18-ACE2 mice infected with SARS-CoV-2 demonstrated severe lung inflammation 5 days post-infection. Vehicle-treated SARS-CoV-2 infected K18-ACE2 mice displayed immune cell infiltrates in large areas of the lung with focal accumulation in the adjacent alveolar spaces and severe thickening of the alveolar wall, whereas mice pre-treated with diABZI-4 were protected from these effects (**Fig. 3K**). Interestingly, diABZI-4 also protected against influenza A (IAV)-induced lethality (**Fig. S3E**) and IAV replication in lung tissue (**Fig. S3F**). Thus, diABZI-4 confers broad protection against respiratory RNA virus infections.

### diABZI-4 promotes myeloid and lymphocyte activation in the lung

**Fig. 4:**
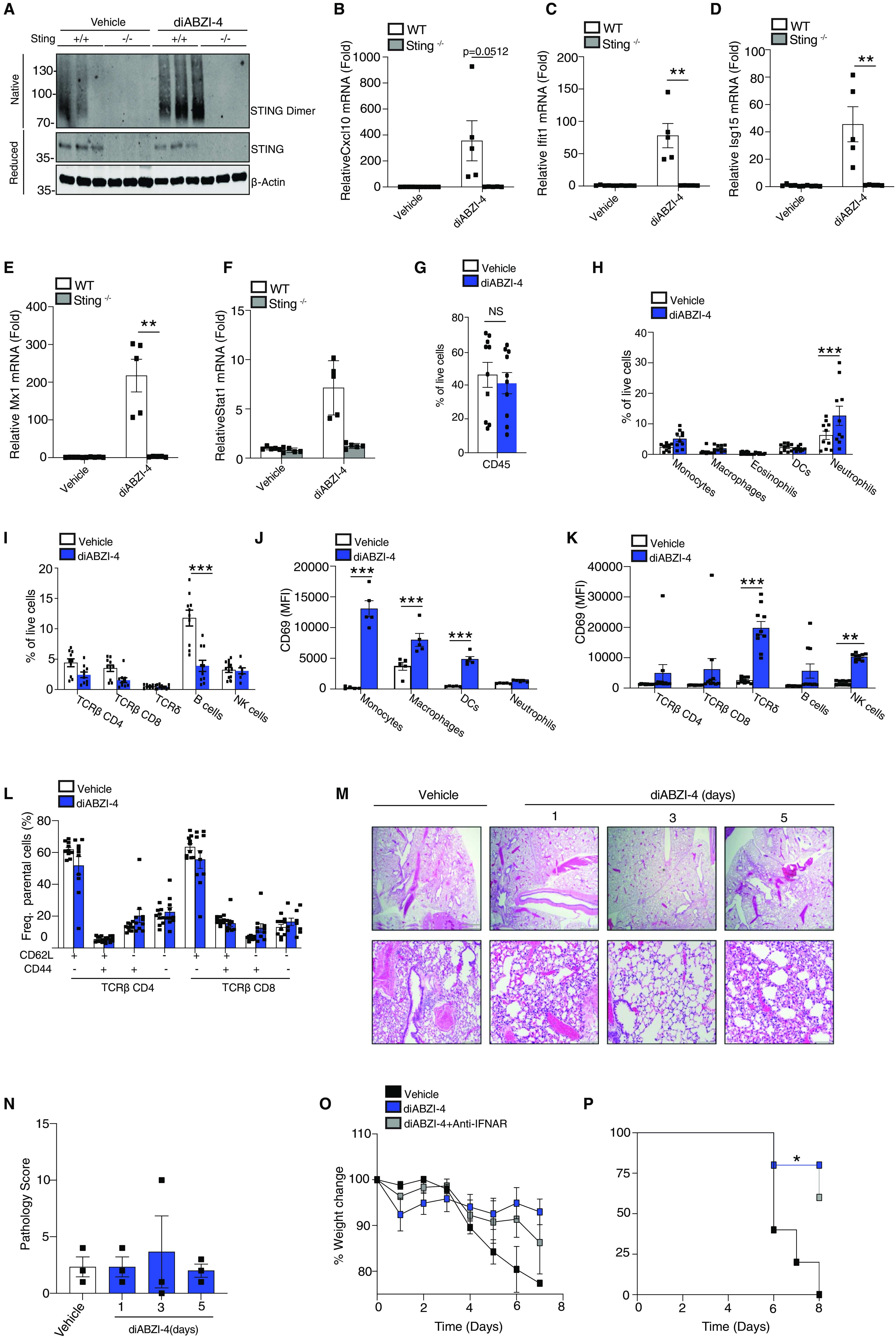
**diABZI-4 mediates protection in an IFN dependent manner (A)** Native immunoblot of STING dimerization in lung tissue from WT and Sting^−/−^ mice administered 0.5mg/kg of diABZI-4 for 3 hours. **(B-F)** QPCR analysis of *Cxcl10*, *Ifit1*, *Isg15*, *Mx1* and *STAT1* mRNA in lung tissue of WT and Sting^−/−^ mice treated intranasally with 0.5 mg/kg diABZI-4 for 3 hours. **(G-L)** Flow cytometry analysis of immune cell populations of PBS perfused, collagenase-digested lung tissue from WT mice treated with 0.5 mg/kg of diABZI-4 for 12 hours (*n=5-10 per group*). Gating strategy is outlined in Fig S5. **(M-N)** Representative images (M) and pathology evaluation (N) of H&E-stained lung sections from mice treated intranasally with 0.5 mg/kg diABZI-4 (*n=3*) or vehicle control (*n=3*) for 1, 2 and 5 days. (**O-P**) Survival (N) and weight loss (O) of K18-ACE2 transgenic mice infected intranasally with SARS-CoV-2 (2.5x10^4^ PFU/mouse) pre-treated intranasally with 150 μg IgG1 (*n=4*), IgG1+diABZI-4 (*n=5*) or anti-IFNAR+diABZI-4 (*n=5*). Error bars show means ± SEM. Data points indicate individual mice. *, *P*< 0.05; **, *P*<0.01; ***, *P*<0.001. (B-F Student’s *t* test; G-L, O two-way ANOVA; P Mantel-Cox survival analysis)

We next sought to investigate the mechanism by which diABZI-4 mediates its protective effects. Intranasal administration of diABZI-4 resulted in the potent oligomerization of STING in the lung (**Fig. 4A**). diABZI-4 also induced the subsequent expression of a large number of genes (**Fig. S4B**) including interferon-stimulated genes such as *Cxcl10* (**Fig. 4B**), *Ifit1* (**Fig. 4C**), *Isg15* (**Fig. 4D**), *Mx1* (**Fig. 4E**) and *Stat1* (**Fig. 4F**) in a STING-dependent manner. Previous reports have demonstrated that STING activation elicits a type I interferon response that stimulates interferon receptor signaling in tumor-resident dendritic cells and leads to antitumor CD8^+^ T cell and NK cell responses (*23*–*25*). In addition, CD8^+^ T cells are essential for inhibition of tumor growth by diABZI based STING agonists (*14*). Thus, we next profiled immune cells in the lung following intranasal delivery of diABZI-4 (**Fig. 4G-L****, Fig. S5A-C**). Except for neutrophils (**Fig. 4H****, Fig. S5A**) and B cells (**Fig. 4I****, Fig. S5B**), no significant changes in overall cell numbers were observed following intranasal treatment with diABZI-4 (**Fig. 4H-J****, Fig. S5A-B)**). Interestingly, diABZI-4 treatment promoted the activation of myeloid cells, γδ T cells and NK cells as evidenced by a significant increase in CD69^+^ cells (**Fig. 4J-K****, Fig. S6A-B**). diABZI-4 did not alter the frequency of central memory and effector T cells (**Fig. 4L****, Fig. S6C**). Despite the antiviral, proinflammatory environment elicited by diABZI-4 in the lung, pathology evaluation of lung tissue 1, 2 and 5 days after intranasal delivery of diABZI-4 showed no gross pathological changes to the lung parenchyma. These data indicate that a single dose strategy is sufficient to eliminate viral infection without any pathological damage to the lung tissue.

### diABZI-4 protects against SARS-CoV-2 infection through IFN-dependent and -independent mechanisms

Type I IFNs play an important role in restricting viral infection. Thus, we next assessed if induction of type I IFNs mediated the protective effects of diABZI-4 against SARS-CoV-2 infection. Intranasal treatment with anti-IFNAR inhibited diABZI-4 induced ISG expression (**Fig. S7A-B**). Anti-IFNAR neutralizing antibody delivered intranasally partially but not completely blocked the protective effects of diABZI-4 in K18-ACE2 mice infected with SARS-CoV-2 (**Fig. 4O-P**). Furthermore, a single dose intranasal dose of diABZI-4 conferred a much greater level of protection against SARS-CoV-2 infection when compared to a single dose of intranasal universal IFN or poly(I:C) **(Fig. S7C-D)**. Universal IFN is a human IFN-alpha A/D hybrid which exhibits activity across multiple species. These data indicate that both IFN-dependent and -independent downstream of STING mediate the protective effects of diABZI-4 in controlling SARS-CoV-2 infection in vivo.

## DISCUSSION

We have identified a host-directed therapy that is efficacious for the treatment of SARS-CoV-2 infection. Pharmacological activation of STING in the lung during SARS-CoV-2 infection elicits a rapid short-lived antiviral response via type I IFNs, NF-κB driven cytokine production and lymphocyte activation resulting in inhibition of viral replication and prevention of severe respiratory disease. Use of diABZI-4 over other immunotherapies such as recombinant IFN offers several significant advantages including cost, enhanced stability, room temperature storage and potential for efficacy at low dose treatments.

SARS-CoV-2 is a respiratory pathogen which can infect ACE2 positive cells in the upper and lower respiratory tract. Type II alveolar cells are readily infected by SARS-CoV-2 and represent approximately 15% of alveolar cells. Type II alveolar cells are essential to produce surfactant proteins and support epithelial barrier integrity, innate immune responses and airway regeneration following lung insult (*26*). iAT2-ALI 3D cultures mimic the human airway and mirror host transcriptional responses to SARS-CoV-2 (*27*). diABZI-4 showed strong inhibition of SARS-CoV-2 replication in iAT2 cells and inhibited SARS-CoV-2-induced cell death of SP2 positive alveolar cells. Thus, the use of diABZI-4 in the human lung is predicted to be effective against SARS-CoV-2.

In vivo, diABZI-4 was efficacious against SARS-CoV-2 prior to and after infection. diABZI-4 administered 12 hours after SARS-CoV-2 infection provided comparable protection to diABZI-4 given as a pre-treatment. The infection of hACE2-transgenic mice with SARS-CoV-2 results in rapid weight loss and morbidity over a period of 6-8 days. Thus, the relative time between infection and peak viral load is extremely short which provides limitations for the use of diABZI-4 to be administered therapeutically. Indeed, the use of other immunotherapies such as intranasal IFN or poly(I:C) are also limited by the rapid kinetics of SARS-CoV-2 infection. Therapeutic utility of multiple doses of universal IFN or poly(I:C) in a hamster model of SARS-CoV-2 was also limited to 24 hours after infection (*28*). While type I IFNs play an essential role in initiating the adaptive immune response to SARS-CoV-2, they alone are not sufficient to control SARS-CoV-2 infection (*29*, *30*). However, other reports in human studies have demonstrated an essential role for type I IFNs in preventing severe COVID-19 (*31*). In support of this some clinical trials have reported positive results for use of IFN in the early stages of COVID-19 (*32*). STING activation triggers type I IFN production which mediates activation of CD8+ T-cell responses. However, in addition to type I IFN responses NF-κB and non-canonical autophagy are also induced downstream of STING (*10*). Indeed, recent studies have identified IFN-independent roles for STING in the control of DNA virus infection and anti-tumor immunity (*33*–*35*). In addition to SARS-CoV-2, diABZI-4 also conferred protection from IAV infection. Thus, the host-directed immune response activated by diAZBI-4 may have broad treatment applications to other respiratory pathogens. Like other immunotherapies, future work will be required to determine the appropriate dose and means of delivery for the use of diABZI-4 in humans. Our study provides molecular and cellular characterization of diABZI-4 in the prevention of SARS-CoV-2 replication, treatment of COVID-19 and highlights its potential use as a treatment for COVID-19 and its potential for the treatment of future pandemics caused by respiratory pathogens in humans.

## MATERIALS AND METHODS

### Study design

The aim of this study was to investigate the antiviral properties of a diABZI-based STING agonist. We investigated the effect of diABZI-4 on human coronavirus infections in cultured lung epithelial cells. Using a human ACE2 transgenic mouse model we determined if the immune responses triggered by STING could be leveraged as an antiviral treatment for SARS-CoV-2 infection. Sample sizes used in each experiment are detailed in the figure legends.

### Biosafety

All study protocols were approved reviewed and approved by Environmental Health and Safety and Institutional review board at University of Massachusetts Medical School prior to study initiation. All experiments with SARS-CoV-2 were performed in a biosafety level 3 laboratory by personnel equipped with powered air-purifying respirators.

### Mice

All animal experiments were approved by the Institutional Animal Care Use Committees at the University of Massachusetts Medical School. Animal were kept in a specific pathogen free (SPF) environment. Hemizygous K18-hACE2 C57BL/6J mice (strain: 2B6.Cg-Tg(K18-ACE2)2Prlmn/J) were obtained from The Jackson Laboratory. cGAS KO mice and STING KO mice were used as previously described (*36*). Animals were housed in groups and fed standard chow diets. Sample sizes used are in line with other similar published studies.

### diABZI-4 treatment

8–12-week-old male and female mice, were anesthetized with isoflurane and administered 0.5mg/kg diABZI-4 intranasally for the indicated times.

### Culture of SARS-CoV-2

T175 flaks of Vero E6 cells were infected with the USA-WA1/2020 (NR-52281; BEI Resources) at an MOI of 0.1 for 48 hours. Supernatants were centrifuged at 450 g for 10 min and aliquoted and stored at -80°C. Virus titer was determined by TCID50 assay in Vero E6 cells.

### Culture of HCoV-OC43

T175 flaks of Vero E6 cells were infected with Hcov-0C43 (NR-52281; BEI Resources) at an MOI of 0.1 for 48 hours. Supernatants were centrifuged at 450 g for 10 min and aliquoted and stored at -80°C. Virus titer was determined by TCID50 assay in Vero E6 cells.

### SARS-CoV-2 infection

8–12-week-old male and female mice were anesthetized with intraperitoneal injection of ketamine (100 mg kg^−1^ body weight) and xylazine (10 mg kg^−1^ body weight). Mice were then infected intranasally with 2.5 × 10^4^ PFU of SARS-CoV-2. Mice were monitored daily for weight loss and survival.

### PR8-influenza A infections

8–12-week-old male and female mice, were anesthetized with isoflurane and intranasally infected with 400 PFU of Influenza A/PR/8/34 (H1N1). Mice were monitored daily for weight loss and survival.

### HSV-1 infections

8–12-week-old male and female mice were anesthetized with intraperitoneal injection of ketamine (100 mg kg^−1^ body weight) and xylazine (10 mg kg^−1^ body weight). Corneas were scratched in a 10 × 10 crosshatch pattern and mice were either inoculated with 2 × 10^5^ PFU of HSV-1 in 5 μl of PBS or mock infected with 5 μl of PBS. Mice were monitored daily for weight loss and assessed for ocular hair loss, eye swelling, hydrocephalus and symptoms related to neurological disease as previously described.

### HSV-1 plaque assay

Vero cells (5x10^5^) were plated in 6-well plates in DMEM containing 10% FCS and 1% penicillin streptomycin. The following day, brains were extracted from mice and homogenized in 1ml of medium. Brains were centrifuged for 10 min at 8000 g. Supernatant was collected and serially diluted 2-fold in DMEM. 500ul of each dilution was added to the Vero cells for 1 hour with gentle shaking. After 1 hour, medium was removed and 2 ml DMEM containing 15 μg/ml purified human IgG (Sigma I4506). Cells were incubated at 37°C for 2 days. Medium was then removed, and cells were fixed in 100% ice cold methanol for 3 min. Plaques were stained with 10% crystal violet for 20 min with gentle shaking, washed and counted.

### SARS-CoV-2 RNA analysis

Two days post infection, mice were euthanized in isoflurane. Lung tissue was placed in a bead homogenizer tube with 1 ml of MEM + 2% FBS. After homogenization, 100 μl of this mixture was placed in 300 μl Trizol LS (Invitrogen) and RNA was extracted with the Direct-zol RNA miniprep kit (Zymo) per the manufacturer’s instructions. Quantification of SARS-CoV-2 RNA levels was performed using the QuantiFast Pathogen RT-PCR kit (Qiagen) and the US Centers for Disease Control and Prevention real-time RT-PCR primer/probe sets for 2019-nCoV_N2 (IDT).

### SARS-CoV-2 viral titer

SARS-CoV-2 and HCoV-OC43 were titered in Vero E6 cells using median tissue culture infectious dose (TCID50). As previously described. Briefly, supernatants from SARS-CoV-2 infected ACE2-A549 cells were collected and 10 replicates per sample were 10-fold serially diluted in Vero E6 cells for 6 days and assessed for the presence of cytopathic effect (CPE). TCID50 was calculated using the Reed and Muench formula.

### cDNA synthesis and real time PCR

Total RNA was extracted from whole lung tissue or cells. 1 μg of RNA was reverse transcribed using the iScript cDNA synthesis kit (Bio-Rad). 5 ng of cDNA was then subjected to qPCR analysis using iQ SYBR Green Supermix reagent (Bio-Rad). Gene expression levels were normalized to TATA-binding protein (TBP) or HPRT. Relative mRNA expression was calculated by a change in cycling threshold method as 2^-ddC(t)^. Specificity of RT-qPCR amplification was assessed by melting curve analysis. The sequences of primers used in this study are listed in Table 1.

**Table 1:** PCR primer sequences.

**Primer name**	**Primer Sequence**
Human IFNβ F	GTC TCC TCC AAA TTG CTC TC
Human IFNβ R	ACA GGA GCT TCT GAC ACT GA
Human CXCL10 F	GTG GCA TTC AAG GAG TAC CTC
Human CXCL10 R	TGA TGG CCT TCG ATT CTG GAT T
Human TNF F	CCT CTC TCT AAT CAG CCC TCT
Human TNF R	GAG GAC CTG GGA GTA GAT GAG
Human HPRT F	ATC AGA CTG AAG AGC TAT TGT AAT GA
Human HPRT R	TGG CTT ATA TCC AAC ACT TCG TG
murine IFNβ F	ATA AGC AGC TCC AGC TCC AA
murine IFNβ R	CTG TCT GCT GGT GGA GTT CA
murine Cxcl10 F	CCA AGT GCT GCC GTC ATT TTC
murine Cxcl10 R	GGC TCG CAG GGA TGA TTT CAA
murine TNF F	CAG TTC TAT GGC CCA GAC CCT
murine TNF R	CGG ACT CCG CAA AGT CTA AG
murine IL6 F	AAC GAT GAT GCA CTT GCA GA
murine IL6 R	GAG CAT TGG AAA TTG GGG TA
murine Isg15 F	CTGTACCACTAGCATCACTGTG
murine Isg15 R	GGTGTCCGTGACTAACTCCAT
murine Ifit1 F	GCCTATCGCCAAGATTTAGATGA
murine Ifit1 R	TTCTGGATTTAACCGGACAGC
murine Mx1 F	AGACTTGCTCTTTCTGAAAAGCC
murine Mx1 R	GACCATAGGGGTCTTGACCAA
murine Stat1 F	TCA CAG TGG TTC GAG CTT CAG
murine Stat1 R	GCA AAC GAG ACA TCA TAG GCA
murine TBP F	GAAGCTGCGGTACAATTCCAG
murine TBP R	CCCTTGTACCCTTCACCAAT
HCoV-OC43-N F	AGGAAGGTCTGCTCCTAATTC
HCoV-OC43-N R	TGCAAAGATGGGGAACTGTGGG
SARS-CoV-2-N F	CTCTTGTAGATCTGTTCTCTAAACGAAC
SARS-CoV-2-N R	GGTCCACCAAACGTAATGCG
Nsp14 F	TGGGGYTTTACRGGTAACCT
Nsp14 R	AACRCGCTTAACAAAGCACTC
SARS-CoV-2 ORF1 F	GAGAGCCTTGTCCCTGGTTT
SARS-CoV-2 ORF1 R	AGTCTCCAAAGCCACGTACG

### NanoString

Total RNA was isolated from whole colon using Aurum^TM^ Total RNA Mini kit (Bio-Rad) and quantitated by a Nanodrop ND-1000 spectrophotometer (ThermoFisher Scientific). 50 ng of RNA was then hybridized with a custom probe set and data was analyzed using the NanoString nSolver analysis system (NanoString technology). Gene expression data was normalized to internal positive and negative controls. Heat map was generated using R-software.

### Cell culture

Human ACE2-A549 and Vero E6 cells were cultured in Dulbecco’s modified Eagle’s medium supplemented with 10% (v/v) fetal bovine serum, 100 U/ml penicillin and 100 μg/ml streptomycin. Human peripheral blood monocyte cell line were cultured in RPMI-1640 medium supplemented with 10% (v/v) fetal bovine serum, 100 U/ml penicillin and 100 μg/ml streptomycin. For isolation of BMDMs, tibias and femurs were removed from wild type mice and bone marrow was flushed with complete DMEM medium. Cells were plated in medium containing 20% (v/v) conditioned medium of L929 mouse fibroblasts cultured for 7 days at 37°C in a humidified atmosphere of 5% CO_2_. Medium was replaced every 3 days.

### Human stem cell differentiation to type II Pneumocyte cells and Air-Liquid-Interface (ALI) cultures

Human pluripotent stem cell-derived Type II pneumocytes (iAT2) were differentiated as previously described (*37*). Briefly, H1 human embryonic stem cells (hESC) were maintained on hESC-qualified Matrigel (Corning, Cat# 354277) coated plates in mTeSR1 media (STEMCELL Technologies, Cat# 85850). hESCs were differentiated to definitive endoderm using the STEMdiff Definitive Endoderm kit (STEMCELL Technologies, Cat# 05110). On day 5, colonies were dissociated into small chunks using gentle dissociation reagent (Gibco, Cat# 07174) and plated at a 1:4 ratio onto growth factor reduced Matrigel (GFR; Corning, Cat# 354230) coated wells in DS/BS media containing complete serum free differentiation media (cSFDM) (*38*) supplemented with 10 μM SB431542 (Tocris Bioscience, Cat# 1614) and 2 μM dorsomorphin (Stemgent, Cat# 04-0024). After 3 days of anteriorization, cells were changed to lung progenitor induction media, containing cSFDM media with 3 μM CHIR99021 (Tocris Bioscience, Cat# 4423), 10 ng/mL rhBMP4 (R&D Systems, Cat# 314-BP), and 100 nM retinoic acid (Sigma, Cat# R2625) for 7 days. On day 15, lung progenitor cells were sorted (FACSAria Fusion) based on high CPM expression and plated cells in 50-100 μl droplets (300-500 cells/μl) using GFR Matrigel matrix. After 20 min of 3D Matrigel matrix solidification, K+DCI media (cSFDM media supplemented with 10 ng/mL rhKGF (R&D, Cat# 251-KG-010), and 50 nM dexamethasone (Sigma, Cat# D4902), 0.1 mM 8-bromoadenosine 30,50-cyclic monophosphate sodium salt (Sigma, Cat# B7880) and 0.1 mM 3-isobutyl-1-methylxanthine (Sigma, Cat# I5879) was added. On day 17.5, media was changed to CK+DCI media (K+DCI media added with 3μm CHIR99021). After 3D alveolosphere formation for 7-10 days, cells were passaged using 2 mg/ml of dispase (Gibco, Cat# 17105-041) and TripLE Express (STEMCELL Technologies, Cat# 12604-013). On day 30-35, cells were sorted again based on high CPM expression, and passaged every 7-10 days in 3D culture format. To further mature iAT2 cells, approximately 100K to 150K cells were plated onto GFR-coated 24-well air-liquid-inserts (Corning, Cat# 3470). Once cells reached confluency (after 2-4 days), media was added only to the basolateral chamber to expose the apical surface to air. After 2 weeks of iAT2-ALI culture maturation, cells were used for infection experiments.

### Immunofluorescence

iAT2-ALI cells were fixed in 4% paraformaldehyde for 20 min at RT. After two PBS washes, cells were incubated with blocking buffer containing 5% donkey serum (Lampire Biological Laboratories, Cat# 7332100) in PBST buffer (0.2% Triton-x-100 in 1X PBS) for 45 min at RT. After blocking, cells were incubated overnight at 4°C in blocking buffer containing primary antibodies against SARS-CoV-2 Spike (Novus Biological, Cat# NBP2-90980G), pro-surfactant protein C (Abcam, Cat # ab90716) and NKX2.1 (Thermo Fisher scientific, Cat # MA5-13961). Next day, cells were washed in PBST buffer 3 times for 5 min each. Cells were incubated with Alexa Fluor conjugated secondary antibodies for 2 hours at RT. After 3 PBST washes, nuclei were stained with Hoechst 33342 (Thermo Fischer Scientific, Cat# H3570) for 7 min and stored in PBS. Images were captured using Nikon Eclipse Ti microscope.

### ELISA

Conditioned media or serum was collected as indicated and mouse or human IFNβ, TNF-α, CXCL10 and IL-6 were quantified by sandwich ELISA (R&D Systems).

### Immunoblotting

For cell lysate analysis cells were lysed directly in (50 mM Tris-HCl, pH 7.4, containing 150 mM NaCl, 0.5% (w/v) IGEPAL, 50 mM NaF, 1 mM Na3VO4, 1 mM dithiothreitol, 1 mM phenylmethylsulfonyl fluoride and protease inhibitor cocktail. For native gel analysis cells were lysed in in NP-40 lysis buffer and stored in native loading buffer. Samples were resolved by SDS-PAGE and transferred to nitrocellulose membranes and analyzed by immunoblot. Immunoreactivity was visualized by the Odyssey Imaging System (LICOR Biosciences). Anti-p-IRF3, anti-p-P65, anti-p-TBK1, anti-LC3A/B and anti-STING antibodies were purchased from Cell Signaling Technology. Anti-β-actin (AC-15; A1978) was purchased from Sigma; anti-mouse IRDye^TM^ 680 (926-68070) and anti-rabbit IRDye^TM^ 800 (926-32211) were from LI-COR Biosciences.

### Histology and immunohistochemistry

Tissue blocks were sectioned at 5 μm thick. For paraffin-embedded tissue, lungs were intratracheally inflated with 10% buffered-formalin and dissected from mice. Tissues were fixed in 4% paraformaldehyde overnight before being processed and embedded in paraffin. Five micrometer thin sections were stained by H&E or PAS in an automated stainer (Leica Autostainer XL). Histomorphology of each H&E slide was evaluated by Applied Pathology Systems at low and high-power field on an Olympus BX40 microscope, and the images were captured with Olympus cellSens Entry software at X4 magnifications. Grading of histology scores were performed by Applied Pathology Systems. Tissue sections were stained with H&E for evaluation of inflammation. IHC staining of PR8-IAV nucleoprotein-1 (NP-1) was performed with anti-influenza A virus nucleoprotein antibody (AA5H; ab20343, Abcam) and detected using horseradish peroxidase-conjugated secondary antibodies after heat-induced antigen retrieval as previously described. Diaminobenzidine was used for detection. Images were captured with a Nikon Ds-Ri2 microscope.

### Flow cytometry

Vehicle– or diABZI-4–treated mice were anesthetized with ketamine and transcardially perfused with PBS until lung tissue turned white in color. Lung tissue was minced and digested in collagenase A (Roche) and 30 μg/ml DNase I (Sigma-Aldrich) in RPMI at 37°C for 45 min. Digested tissue was filtered through a 70 μm filter and washed and resuspended in MACS buffer. Isolated lung mononuclear cells were stained with anti-CD45.2 BV650, anti-CD11b BV510, anti-Ly6C APC, anti-TCRß PerCP-Cy5.5, anti-CD8 Alexa 700, anti-CD11c eF450, anti-CD64 BV711, anti-Ly6G PE-Cy7, anti-Ly6G FITC, anti-CD3 PerCP-ef710, anti-CD4 APC-Cy7, anti-TCRẟ PE, anti-B220 PE-Cy5, anti-CD326 APC, anti-CD45.1 APC-Cy7. Cells were acquired on a Cytek Aurora cytometer. Flow cytometry analysis was done with the FlowJo software.

### CD14^+^ monocyte isolation

PBMC were isolated from whole blood of consenting donors. Blood was diluted 1:1 in sterile PBS and layered over 15 mls of Lymphoprep. Blood was spun at 450*g* with no brake. The interphase was transferred to a fresh tube using a Pasteur pipette and washed twice in PBS. Red blood cells were lysed in red blood cell lysis buffer for 10 min at room temperature. Cells were washed once more in PBS and counted. CD14^+^ monocytes were isolated using human CD14 magnetic microbeads (Miltenyi), washed twice in ice-cold macs buffer and plated in RPMI medium.

### Ethics

All animal studies were performed in compliance with the federal regulations set forth in the Animal Welfare Act (AWA), the recommendations in the Guide for the Care and Use of Laboratory Animals of the National Institutes of Health, and the guidelines of the UMass Medical School Institutional Animal Use and Care Committee. All protocols used in this study were approved by the Institutional Animal Care and Use Committee at the UMass Medical School (protocols A-1633).

### Statistical analysis

For comparisons of two groups two-tailed Students’ *t* test was performed. Multiple comparison analysis was performed using two-way ANOVA. 3 to 16 mice were used per experiment, sufficient to calculate statistical significance, and in line with similar studies published in the literature. Randomization and blinding were not performed.

## Supplementary Materials

immunology.sciencemag.org/cgi/content/full/6/59/eabi9002/DC1 Supplementary Methods Fig. S1 diABZI-4 activates STING in murine macrophages Fig. S2 cGAS and STING deficient mice are highly susceptible to HSE Fig. S3 diABZI-4 inhibits Influenza A infection. Fig. S4 diABZI-4 induces an antiviral gene expression response in the lung. Fig. S5 Absolute lung cell numbers and flow cytometry gating strategy Fig. S6 Representative CD69 positive flow cytometry plots Fig. S7 Anti-IFNAR inhibits diABZI-4 induced type I IFN signaling in the lung Data file S1. Raw data file (Excel) Reproducibility checklist

## Notes

**Note added in proof:** Another complementary study by Li et al has shown that diABZI STING agonists protect against SARS-CoV-2 infection (*39*).
